# Smurf2 as a novel mitotic regulator: From the spindle assembly checkpoint to tumorigenesis

**DOI:** 10.1186/1747-1028-4-14

**Published:** 2009-07-07

**Authors:** Evan C Osmundson, Dipankar Ray, Finola E Moore, Hiroaki Kiyokawa

**Affiliations:** 1 Department of Molecular Pharmacology and Biological Chemistry, Northwestern University Feinberg School of Medicine, 303 E. Chicago Avenue, Chicago, IL 60611, USA; 2 Robert H. Lurie Comprehensive Cancer Center, Northwestern University Feinberg School of Medicine, 303 E. Superior Street, Chicago, IL 60611, Chicago, IL 60611, USA; 3 Department of Biochemistry and Molecular Genetics, University of Illinois College of Medicine, 900 S. Ashland Avenue, Chicago, IL 60607, USA; 4 Division of Cell Biology and Physiology, Indian Institute of Chemical Biology (CSIR Institute), 4, Raja S.C. Mullick Road, Jadavpur, Kolkata 700 032, India

## Abstract

The execution of the mitotic program with high fidelity is dependent upon precise spatiotemporal regulation of posttranslational protein modifications. For example, the timely polyubiquitination of critical mitotic regulators by Anaphase Promoting Complex/Cyclosome (APC/C) is essential for the metaphase to anaphase transition and mitotic exit. The spindle assembly checkpoint prevents unscheduled activity of APC/C-Cdc20 in early mitosis, allowing bipolar attachment of kinetochores to mitotic spindle and facilitating equal segregation of sister chromatids. The critical effector of the spindle checkpoint, Mitotic arrest deficient 2 (Mad2), is recruited to unattached kinetochores forming a complex with other regulatory proteins to efficiently and cooperatively inhibit APC/C-Cdc20. A weakened and/or dysfunctional spindle checkpoint has been linked to the development of genomic instability in both cell culture and animal models, and evidence suggests that aberrant regulation of the spindle checkpoint plays a critical role in human carcinogenesis. Recent studies have illuminated a network of both degradative and non-degradative ubiquitination events that regulate the metaphase to anaphase transition and mitotic exit. Within this context, our recent work showed that the HECT (Homologous to E6-AP C-terminus)-family E3 ligase Smurf2 (Smad specific ubiquitin regulatory factor 2), known as a negative regulator of transforming growth factor-beta (TGF-β) signaling, is required for a functional spindle checkpoint by promoting the functional localization and stability of Mad2. Here we discuss putative models explaining the role of Smurf2 as a new regulator in the spindle checkpoint. The dynamic mitotic localization of Smurf2 to the centrosome and other critical mitotic structures provides implications about mitotic checkpoint control dependent on various ubiquitination events. Finally, deregulated Smurf2 activity may contribute to carcinogenesis by perturbed mitotic control.

## The spindle assembly checkpoint

The spindle assembly checkpoint is a complex signal transduction cascade that inhibits the metaphase to anaphase transition in the presence of unattached or untensed kinetochores. It incorporates a molecular surveillance mechanism whereby a single unattached kinetochore is sufficient to initiate a global/cell-wide inhibitory signal to prevent the biochemical events driving anaphase onset. The critical downstream target of the spindle checkpoint is APC/C which, in association with its activator Cdc20, is responsible for the rapid ubiquitin-dependent degradation of mitotic regulatory proteins such as Securin, an inhibitor of sister chromatid segregation, Cyclin B, and Aurora B kinase. In early mitosis, the checkpoint protein Mad2 is recruited to sites of unattached kinetochores in association with Mad1 (Mitotic arrest deficient 1), Bub1 (Budding uninhibited by benzimidazoles 1 homolog) and other components of the Mitotic Checkpoint Complex (MCC). The association of Mad2 with various components of the MCC at the kinetochore is thought to induce a dramatic conformational change of Mad2 allowing for the tight binding between Cdc20 and Mad2 and the inhibition of both kinetochore-associated and cytoplasmic APC/C. This prevents premature APC/C activity which would trigger mitotic exit and the development of numerical chromosomal abnormalities. Implicit in this mechanism is the requirement for reversibility, allowing for the rapid activation of APC/C once all kinetochores are securely attached to spindle microtubules. During spindle checkpoint activation, Mad2 undergoes a dramatic conformational change from an inactive to an active conformer capable of binding to and inhibiting Cdc20-associated APC/C. While the evidence for the two state model of Mad2 is abundant, the spontaneous conversion of Mad2 from an inactive form to an active, inhibitory conformation takes several hours, evoking the need for a catalytic mechanism [1]. The precise mechanisms allowing for both the rapid reversibility and the global nature of the inhibitory signal remain controversial, and leading hypotheses have been recently reviewed [2,3].

It has been known for some time that rapid APC/C-mediated polyubiquitination and degradation of diverse substrates drives anaphase onset and the segregation of sister chromatids. Recent work, however, has implicated non-canonical roles for ubiquitination in the regulation of the spindle checkpoint. Stegmeier, Reddy and others have provided evidence for an antagonistic cycle of ubiquitination and deubiquitination of coactivator Cdc20 in early mitosis which regulates the assembly of Cdc20-Mad2 complexes, and thereby APC/C activation and spindle checkpoint activity [4,5]. Available evidence supports that USP44-mediated deubiquitination stabilizes the Cdc20-Mad2 complex by removing ubiquitin conjugates from Cdc20 that would otherwise promote disassembly of Cdc20-Mad2. Importantly, this work strongly implies that the non-degradative ubiquitination of Cdc20 is a prerequisite for the onset of timely APC/C activity. This must be reconciled with recent evidence presented by Pagano and Pines groups who argue that during spindle checkpoint activation, a significant proportion of Cdc20 is rapidly polyubiquitinated and degraded, perhaps in a Mad2-dependent manner, a process which may be essential for spindle checkpoint activation [6,7]. Taken together, these studies suggest that an intricate network of both degradative and non-degradative ubiquitination events regulates mitotic progression. Adding to this complexity is our recent study indicating the activity of Smurf2, a HECT family E3 ligase, in the stability of Mad2 and the function of the spindle checkpoint [8].

## Smurf2 targets and expanding array of diverse substrates

Like all HECT domain-containing ubiquitin ligases, Smurf2 has a catalytic activity as a single polypeptide. It contains an N-terminal C2-calcium phospholipid binding domain, three WW domains within a flexible linker region, followed by a C-terminal HECT domain responsible for its catalytic activity. Smurf2 was originally characterized as a negative regulator of the TGF-β signaling pathway by targeting receptors, signaling intermediates, and other pathway-specific transcription factors for degradation [9-11]. Recent studies have uncovered new roles and substrates for this E3 beyond the canonical TGF-β signaling pathway. For example, Smurf2 targets the Guanine-Trinucleotide Phosphate hydrolase Rap1B [12], the RING (*Really Interesting New Gene-*domain) protein RNF11 [13], the Runt domain transcription factors Runx2 [14] and Runx3 [15], Beta-catenin [16], and most recently reported, its paralog Smurf1 [17]. Through its role as a regulator of TGF-β mediated transcriptional events, Smurf2 has been tangentially implicated in the control of the cell cycle, but direct evidence implicating Smurf2 in cell cycle control was lacking until recently.

## Smurf2 is a novel mitotic regulator

The first clue that Smurf2 could play a more direct role in cell cycle control came with the evidence that Smurf2 itself is a cell cycle regulated protein. In synchronized HeLa cells, Smurf2 protein levels are lower in late G1 and early S and peak in late G2 and early mitosis. Whereas previous studies had localized Smurf2 to non-clathrin containing, caveolin- and EEA1-positive endosomes [18], our subsequent analyses localized Smurf2 to the centrosome by both immunocytochemical [8] and biochemical means (E. Osmundson, H. Kiyokawa unpublished data). Furthermore, Smurf2 exhibits a highly dynamic localization pattern throughout mitosis, moving from centrosomes in late prophase and metaphase, to the mitotic midzone in anaphase, and ultimately to the midbody in telophase. This localization pattern is reminiscent of, though not identical to, chromosomal passenger proteins (i.e. survivin and Aurora B) which have well established roles in mitotic regulation [19]. Consistent with this notion, our experiments have shown that Smurf2 is required for normal mitotic progression, with acute depletion of Smurf2 in mammalian cells leading to multinucleation and cytokinesis failure. Smurf2-depleted cells often initiate anaphase in the presence of unaligned chromosomes. Further, following chromatin condensation in prophase, Smurf2-depleted cells frequently fail to form a discernable metaphase plate (i.e. chromosomal congression defects) and exit mitosis as a bi- or multi-nucleated cell (i.e. mitotic failure). These phenotypes suggest that Smurf2 plays a role in ensuring proper alignment of chromosomes at metaphase, a process controlled by the spindle assembly checkpoint.

Cells depleted of spindle checkpoint proteins such as BubR1 (Bub1-related kinase), Mad2, or USP44 demonstrate mitotic defects, and fail to arrest in early mitosis in response to spindle toxins like nocodazole or taxol. Similarly, Smurf2-depleted cells also fail to arrest in prometaphase when exposed to nocodazole, displaying premature degradation of the APC/C-Cdc20 substrates, i.e., Securin, Cyclin B, and Aurora B. Co-depletion of Smurf2 and Cdc20 can restore protein levels of these substrates, confirming premature activation of APC/C. Therefore, Smurf2 is required for the spindle assembly checkpoint-mediated APC/C inhibition. Anaphase onset is accelerated in Smurf2-depleted cells, which is analogous to Mad2- or BubR1-depleted cells. Importantly, Mad2 protein levels are significantly decreased and kinetochore localization of Mad2 is virtually undetectable in Smurf2-depleted cells. In contrast, levels and localization of BubR1 are essentially unaffected by Smurf2 depletion. These data suggested that Smurf2 functions upstream of Mad2 in spindle checkpoint control.

## Smurf2-mediated control of Mad2 stability

The regulation of cellular Mad2 levels has been described primarily in terms of transcriptional control, with recent studies demonstrating that Mad2 is a transcriptional target of E2F, BRCA1, Myc, and most recently REST [20-23]. While Mad2 has been shown to be regulated by phosphorylation [24], other potential forms of post-transcriptional modification and regulation of Mad2 have not been thoroughly characterized. Smurf2-mediated control of Mad2 appears to occur at the level of Mad2 protein stability [8]. While there is no change in Mad2 mRNA levels, an increase in polyubiquitin-conjugated Mad2 protein is observed in Smurf2-depleted cells, which is further increased by proteasome inhibition. These observations suggest that Smurf2 normally functions as a suppressor of ubiquitin-dependent degradation of Mad2. Interestingly, Smurf2 may also influence the kinetochore localization of Mad2, since forced overexpression of Mad2 in Smurf2-depleted cells results in a failure of exogenous Mad2 in localizing to kinetochores in the presence of nocodazole.

The role of Smurf2 in the spindle checkpoint appears to be mediated, at least partly, through its activity as an E3 ubiquitin ligase. Cells expressing a catalytically inactive Smurf2 mutant (Smurf2-C716A) also exhibit mitotic defects similar to Smurf2-depleted cells, together with premature activation of APC/C, indicating spindle checkpoint dysfunction.

## Two models of Smurf2-mediated control of Mad2 and the Spindle Assembly Checkpoint

**Figure 1:**
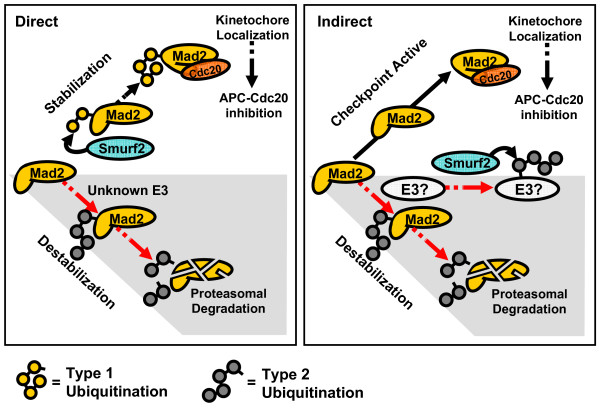
**Putative models depicting the role of Smurf2 in Mad2 stabilization and spindle checkpoint integrity**.

Based on available evidence we propose two models explaining the role of Smurf2 in mitotic checkpoint control (Figure 1). Both of these models build upon evidence suggesting that the role of Smurf2 in the spindle checkpoint is dependent upon its ubiquitin ligase activity. The first model, hereafter designated the direct model, proposes that Mad2-directed E3 ligase activity of Smurf2 functionally antagonizes a yet to be identified E3 ligase which would otherwise promote the polyubiquitination and degradation of Mad2 in various contexts. Given the established importance of the active (APC/C inhibitory) conformer of Mad2 for mitotic checkpoint function, ongoing surveillance for a match between the conformational state of Mad2 and cellular conditions/checkpoint status should be critical for checkpoint function. For example, due to the partially autocatalytic mechanism of Mad2 conformational change, the presence of the non-inhibitory Mad2 conformer above a critical threshold during checkpoint activating conditions would be highly deleterious. Consistent with the direct model, it is plausible that Smurf2 mediated attachment of mono-ubiquitin or non-Lys48 linked (non-degradative) ubiquitin chains (e.g. Lys63 linkage) promotes the transition to the APC/C inhibitory conformer of Mad2 during checkpoint activation. Such a posttranslational modification may stabilize the hypothesized intermediate structural state of Mad2 [25] facilitating the structural rearrangements associated with Mad2 conformational activation. In the absence of adequate Smurf2 E3 ligase activity, the rapid degradation of Mad2 during mitotic progression may be an adaptive cellular response in an attempt to eliminate "misfolded" or non-inhibitory Mad2, a condition that is maximized during strong experimental knockdown of Smurf2. In support of this notion, the expression of a Mad2 mutant incapable of assuming the inhibitory conformation results in inactivation of the spindle checkpoint [1,26,27]. Such a model also predicts that differences in the stability of Mad2 conformers could exist depending on the activation status of the spindle checkpoint. To our knowledge this possibility has not been directly addressed. In the direct model, the direct ubiquitination of Mad2 by Smurf2 would also presumably mobilize the appropriate localization of Mad2 to unattached mitotic kinetochores. It is of interest that a recent report described the ubiquitination of the chromosomal passenger protein survivin occurs via both Lys48- and Lys63-linkages. The authors demonstrate that it is the assembly and disassembly of non-degradative Lys63 linked ubiquitin chains that appear to be critical for its association with mitotic kinetochores [28]. A similar mechanism may be at play with Mad2, whereby regulated attachment of distinct ubiquitin linkages would specify alternative fates of Mad2 providing yet another layer of checkpoint control.

In the second model, hereafter referred to as the indirect model, Smurf2 catalyzes the ubiquitin-dependent degradation of an unidentified E3 ligase (or its critical cofactor) that promotes consititutive polyubiquitination of Mad2. In this way, mitotic Smurf2 activity would antagonize the action of this unknown E3 ligase, thereby allowing for stability and proper localization of Mad2 and a functional spindle checkpoint. Conditions leading to decreased levels or activity of Smurf2 would upregulate the activity of this E3 ligase, targeting Mad2 for polyubiquitination and degradation. The implications of the indirect model are best appreciated in context of what is known about the kinetics of Mad2 conformational interconversion. The spontaneous reverse transition from active (inhibitory) to inactive(non-inhibitory) Mad2 conformation is six-fold slower than the forward reaction (which is already unusually slow at ~9 hrs), owing partly to the thermodynamically favored inhibitory conformation [1]. While p31comet-dependent mechanisms exist to block the conversion of inactive to active Mad2 in aggregate [29-31], to our knowledge no mechanisms have been described to catalyze the conversion of the active conformer of Mad2 back to inactive conformer, an energetically costly process. Alternatively, it may be more efficient for the cell to simply bypass this problem through rapid ubiquitin-dependent degradation of the inhibitory Mad2 conformer once the spindle checkpoint has been satisfied, allowing for swift and decisive anaphase onset. Given that kinetochore-microtubule interaction is a stochastic process, this would occur locally on a kinetochore by kinetochore basis. In support of this view, most Mad2 exists in the non-inhibitory conformation in interphase cells [1], and the inhibitory conformation of Mad2 is unlikely to exist as a monomeric form uncomplexed with Mad2, Cdc20, or Mad1 [32]. Viewed in this light, Smurf2 would target the activity of this E3 ligase in early mitosis at unattached kinetochores. As kinetochores become securely attached to mitotic spindle, Smurf2 activity could be locally downregulated or it could be sequestered from the vicinity, which would allow for the local ubiquitin-dependent degradation of the inhibitory conformer of Mad2.

It will be interesting to determine if a direct causal relationship exists between the apparent mislocalization and instability of Mad2 in Smurf2-depleted cells. Recent work has emphasized the importance of localization-dependent activity of ubiquitin ligases (i.e. APC/C) and other participants of the ubiquitin proteasome system [33]. It is plausible that the destiny of Mad2, either degradation or APC/C inhibition, could be entirely dependent upon matching subcellular localization with mitotic state/phase, and that Mad2 is simply degraded when it is mislocalized. Thus the enhanced ubiquitin-dependent degradation of Mad2 observed in Smurf2-depleted cells could be entirely a result of being in the wrong place at the wrong time. The common thread linking these two models is the unknown E3 ligase regulating the degradation of Mad2, whose identity should be determined by future studies.

## A broader role for Smurf2 in mitosis

While defective spindle checkpoint function is likely the major contributor to the mitotic defects in Smurf2 depleted cells, the localization pattern of Smurf2 throughout mitosis suggests that Smurf2 plays a role downstream of the metaphase to anaphase transition. A number of centrosomal proteins, including centriolin and survivin, in association with other members of the chromosomal passenger complex, undergo traffic to the mitotic midzone during anaphase and the midbody at telophase, executing unique roles presumably at each of their successive destinations. Furthermore, the individual depletion of many of these proteins results in a failure of cytokinesis and multi-nucleation possibly in spindle checkpoint-independent manners. Thus, it is likely that post-translational modification of Smurf2 or its association with different cellular factors orchestrates the complex trafficking of Smurf2 to various mitotic structures during mitosis. Current studies are underway to examine the possibility of mitosis-specific, perhaps spindle checkpoint-independent roles of Smurf2.

The centrosome acts as a physical platform for the integration of multicomponent signaling cascades throughout various phases of the cell cycle. It is noteworthy that Smurf2 is detected at the centrosome throughout the cell cycle, and studies thus far have only tangentially addressed a possible role for Smurf2 at the centrosome. Smurf2 depleted cells frequently display both numerical and structural centrosomal abnormalities although this could be explained by mitotic failure after normal centrosomal duplication cycles. Furthermore, several players in the spindle assembly checkpoint (e.g. BubR1, Cdc20, Mad2, and now Smurf2) convene at the centrosome in early mitosis, the significance of which remains obscure.

It is also of interest that Smurf1 controls cell polarity by targeting RhoA for proteasomal degradation [34], and the critical role for RhoA in cytokinesis has been established [35]. A recent paper by Fukunaga et. al reports that Smurf2 can promote the degradation of Smurf1 to increase RhoA signaling and promote metastatic progression [17]. While most previous studies have shown that Smurf1 regulates RhoA activity locally at cellular protrusions, it is plausible that Smurf1 participates in control of RhoA signaling during cytokinesis. Although our group did not observe any appreciable effect of Smurf1 depletion on mitosis or cytokinesis, these experiments do not rule out the possibility that Smurf2-mediated control of Smurf1 regulates RhoA activity during mitotic exit. It remains to be determined whether Smurf2 is involved, either directly or indirectly, in the regulation of RhoA during cytokinesis.

## Implications for deregulation of Smurf2 during tumorigenesis

Genomic instability and aneuploidy are well established properties of cancer, which are often associated with poor prognosis of patients. It is clear that a defective spindle checkpoint can promote genomic instability and aneuploidy at the cellular level. Moreover, recent *in vivo *studies have examined whether aneuploidy secondary to mitotic checkpoint dysfunction is a predisposing event in cancer development [36]. For example, Mad2 haploinsufficiency causes premature anaphase onset and chromosomal instability, which is consistent with the higher susceptibility of Mad2-heterozygous mice to lung tumorigenesis [37]. On the other hand, transgenic mice with inducible Mad2 overexpression also develop a variety of tumors [38], which is consistent with Mad2 overexpression observed in a wide range of human cancers [39]. Furthermore, mouse models with Bub1 hypomorphism [40] or Mad1 haploinsufficiency [41] provide further evidence that misregulation of the spindle assembly checkpoint promotes *in vivo *tumorigenesis through the development of chromosomal instability. Similarly, deregulated Smurf2 activity beyond functionally optimal ranges may promote cancer development with genomic instability. Consistent with this notion, we have recently observed that not only Smurf2 depletion but also forced expression of wild-type Smurf2 to exceedingly high levels can perturb the spindle checkpoint and ultimately cause multinucleation (E. Osmundson and H. Kiyokawa, unpublished observations). This is analogous to the phenotypes of forced overexpression of Mad2 both *in vitro *and *in vivo*.

Previously, Smurf2 has been functionally implicated in tumorigenesis through its role as a negative regulator of growth inhibitory TGF-β signaling. Smurf2 has been shown to be upregulated in esophageal carcinomas [42], and a recent study by Jin et. al, employing a breast cancer tissue array, showed that Smurf2 is upregulated in many breast cancer tissues [43]. Based on their functional analyses they concluded that Smurf2 activity contributes to metastatic progression, yet likely in a manner independent of TGF-β signaling. The *Oncomine *database of gene expression profiles  also show increased expression of Smurf2 is observed in breast, ovary, brain and some other cancers, analogous to increased Mad2 expression. Our recent work highlighting the role of Smurf2 upstream of Mad2 stability control provides new insight into roles for Smurf2 in human carcinogenesis. Viewed in this light, the overexpression of Smurf2 could provide a double-hit to cells undergoing the process of carcinogenesis; by rendering cells unresponsive to the tumor suppressive TGF-β signals, and by promoting spindle checkpoint dysfunction and the genomic instability characteristic of malignant phenotypes. More mechanistic studies are needed to establish the role of deregulated Smurf2 actions in carcinogenesis.

## Conclusion

**Figure 2:**
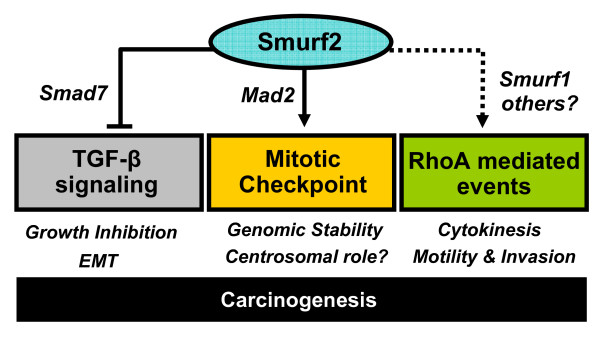
**The roles of Smurf2 in regulating multiple, interrelated cellular processes**. TGF-beta signaling/dysfunction, spindle checkpoint/dysfunction, and possible RhoA-mediated alterations in cytokinesis and cell motility (metastasis).

Our understanding of the spindle assembly checkpoint has matured considerably over the past decade, owing to the refinement of techniques to interrogate the complex enzymatic machinery involved. Still, mechanisms accounting for the robustness of the checkpoint as well as those allowing for its rapid reversal need to be clearly defined. Current evidence suggests that the dynamic nature of checkpoint signaling balances on an axis of ubiquitination and antagonistic deubiquitination of Cdc20 [4,5], and on checkpoint-associated Mad2 conformational change [2]. Although it is clear that spindle checkpoint signaling is initiated at the kinetochore, subsequent spatiotemporal control of these two aspects of checkpoint signaling remains obscure. Our recent data demonstrating that the HECT E3 ligase Smurf2 participates in Mad2 posttranslational control and localization sheds light on previously unappreciated complexities of the spindle checkpoint involving the localization and stability of Mad2 [8]. In addition to its role as a regulator of the spindle checkpoint, the localization of Smurf2 to other critical mitotic structures implies its participation in other aspects of mitotic control, perhaps occurring well before or after the metaphase to anaphase transition. The juxtaposition of mitotic Smurf2 functions with its established role as a negative regulator of TGF-β signaling may provide a mechanism for the heritability of spindle checkpoint-driven epigenetic changes across mitosis into early phases of the next cell cycle. Further experiments are needed to delineate how the mitotic and non-mitotic roles of Smurf2 are integrated throughout the cell cycle (Figure 2). Finally, previous studies focusing on TGF-β associated functions of Smurf2 suggested that Smurf2 misregulation may contribute to tumorigenesis. The role of Smurf2 in Mad2 regulation and spindle checkpoint control provides a new theoretical basis for tumorigenesis occurring through Smurf2 misregulation, and these mechanisms should be clarified by ongoing *in vivo *analysis. Given the expanding role of the spindle checkpoint as a chemotherapeutic target, it is of continued interest to further define the mechanisms of spindle checkpoint control and with it the potential for revealing novel anti-cancer therapeutic targets.

## Competing interests

The authors declare that they have no competing interests.

## Authors' contributions

ECO and HK wrote the manuscript. DR and FEM significantly contributed to designing studies and establishing concepts. All authors read and approved the final manuscript.
